# Case Report: Anti-PL-12 Antisynthetase syndrome complicated by natural killer/T cell non-Hodgkin’s lymphoma

**DOI:** 10.3389/fimmu.2026.1761119

**Published:** 2026-04-24

**Authors:** Kun Li, Mei-E Liang, Yang Liu, Qian-Yu Guo, Ke Xu

**Affiliations:** Third Hospital of Shanxi Medical University, Shanxi Bethune Hospital, Shanxi Academy of Medical Sciences, Tongji Shanxi Hospital, Taiyuan, China

**Keywords:** antisynthetase syndrome, ILD, natural killer/T-cell lymphoma, non-Hodgkin’s lymphoma, PL-12

## Abstract

Antisynthetase syndrome (ASS) is a systemic autoimmune disorder classified as a subtype of the idiopathic inflammatory myopathies (IIM). The condition is defined by the presence of mutually exclusive autoantibodies directed against an aminoacyl-tRNA synthetase along with typical clinical manifestations, including myositis, Raynaud’s phenomenon, arthritis, skin lesions such as mechanic hands, or interstitial lung disease (ILD). Anti-synthetase syndrome associated interstitial lung disease (ASS-ILD) can range from mild forms to rapidly progressive disease, which may lead to chronic pulmonary damage if misdiagnosed or inadequately treated. Patients with IIM carry an increased risk of developing neoplasms, most commonly adenocarcinoma, but not lymphoma or other hematologic malignancies. However, data on the association between ASS and malignancy remain very limited. We report the case of a patient with anti-PL-12 antisynthetase syndrome who subsequently developed NK/T-cell non-Hodgkin lymphoma, supplemented by a review of the pertinent literature.

## 1 Introduction

Antisynthetase syndrome is an autoimmune condition characterized by the presence of autoantibodies directed against an aminoacyl transfer RNA synthetase (aaRS). To date, eight autoantibodies that target these aaRS and are associated with ASS have been identified: anti-PL-7, anti-PL-12, anti-EJ, anti-OJ, anti-KS, anti-Zo, and anti-HA (1). Among these, anti-alanyl-tRNA synthetase (anti-AlaRS), also known as anti-PL-12, is relatively uncommon and is more closely associated with ILD than with other features of the antisynthetase syndrome (2). Moreover, it has been observed that up to 50% of ASS cases are positive for the anti-Ro52 antibody. Patients who are anti-Ro52 positive may be associated with a higher prevalence of ILD (3). According to the latest EULAR clinical practice guidelines, patients with inflammatory myopathy-associated interstitial lung disease (IIM-ILD) should receive prompt immunosuppressive therapy. Moreover, the use of combination therapy involving immunosuppressants and glucocorticoids is suggested, including calcineurin inhibitors, rituximab, Janus kinase inhibitors, and intravenous immunoglobulin (IVIG) (4). Immunosuppressive agents achieve their therapeutic effects while simultaneously compromising the inherent immune surveillance and defense mechanisms, thereby increasing susceptibility to infections and neoplasms.

Regarding specific cancer types associated with ASS, available data indicate that solid tumors are predominant while hematologic malignancies are uncommon. A study comparing long-term outcomes in 75 anti-Jo-1-positive patients and 20 anti-PL-7/PL-12-positive patients with ASS found that 13.3% of anti-Jo-1-positive patients developed cancer, whereas only 1 of 20 patients with anti-PL-7 or PL-12 antibodies (5%) was diagnosed with malignancy (5). Additionally, among 15 anti-PL-7-positive ASS patients, 6.7% were subsequently found to have neoplasms (6). A 4-year follow-up study observed malignancy in 28.6% of anti-OJ-positive patients (7). The elevated cancer risk in patients with ASS is probably multifactorial, driven not by a single factor such as immunosuppressive treatment, but by a complex interaction of disease-related chronic inflammation, autoantibody cross-reactivity, defective immune surveillance, and host factors.

## 2 Case presentation

A 55-year-old female presented with a 7-year history of myalgia, muscle weakness, and shortness of breath, along with pancytopenia identified one month prior.

In 2017, she sought medical attention for facial, neck, and dorsal hand rashes, accompanied by periorbital erythema and edema, myalgia, muscle weakness, cough, sputum production, and fever with a maximum temperature of 38 °C. Laboratory studies revealed elevated muscle enzymes and anti–PL-12 antibody. Chest CT demonstrated interstitial lung changes with inflammatory exudates. She was diagnosed at another institution with anti–PL12 antibody-positive ASS and ASS-ILD. Treatment was initiated with prednisone acetate (50 mg daily), cyclophosphamide (100 mg daily), tacrolimus (0.5 mg twice daily), and trimethoprim-sulfamethoxazole (TMP-SMZ, TMP80mg and SMZ400mg daily). During outpatient follow-up, her medication regimen was gradually tapered over six months to maintenance doses of prednisone acetate (5 mg daily), cyclophosphamide (50 mg daily), tacrolimus (0.5 mg twice daily), and trimethoprim-sulfamethoxazole (TMP-SMZ, TMP80mg and SMZ400mg daily). Her condition remained relatively stable throughout the maintenance period, except for one transient rash recurrence before the current admission.

Seven years after her diagnosis, she experienced an acute onset of fever (peak temperature 38.8 °C), accompanied by cough, sputum production, abdominal pain, and diarrhea. Laboratory tests from another hospital revealed the following: white blood cell count 3.51×10^9^/L (normal range 3.5-9.5×10^9^/L), hemoglobin 134 g/L (normal range 115-150g/L), platelet count 101×10^9^/L (normal range 125-350×10^9^/L), C-reactive protein 82.04 mg/L (normal range <6 mg/L), and NT-proBNP 2591 pg/mL (normal range 0–125 pg/mL). The creatine kinase, creatine kinase-MB, lactate dehydrogenase, and hydroxybutyrate dehydrogenase were within the normal range. Respiratory pathogen testing was positive for Influenza A virus and Mycoplasma pneumoniae. EBV DNA was detected at 2.25×10^4^ copies/mL, and plasma next-generation sequencing identified 210,892 sequences for Epstein-Barr virus. A chest CT scan showed interstitial pneumonia, along with multiple pulmonary nodules and mass-like opacities in both lungs. A bronchial biopsy with immunohistochemical staining demonstrated extensive interstitial lymphocytic infiltration, and the findings were interpreted as an inflammatory process.

Due to the presence of both a pulmonary infection and cardiac dysfunction, her immunosuppressive therapy was discontinued. She was started on intravenous methylprednisolone (40 mg daily), ganciclovir (250 mg twice daily), fluconazole (0.2 g daily), and human immunoglobulin (20 g daily, for 3 days). Additional treatment included cefoperazone/sulbactam for infection and nesiritide for heart failure management. Following this regimen, her fever resolved without recurrence, and her cough and sputum production improved. During hospitalization, she developed left periorbital pain without noticeable discharge.

**Figure 1:**
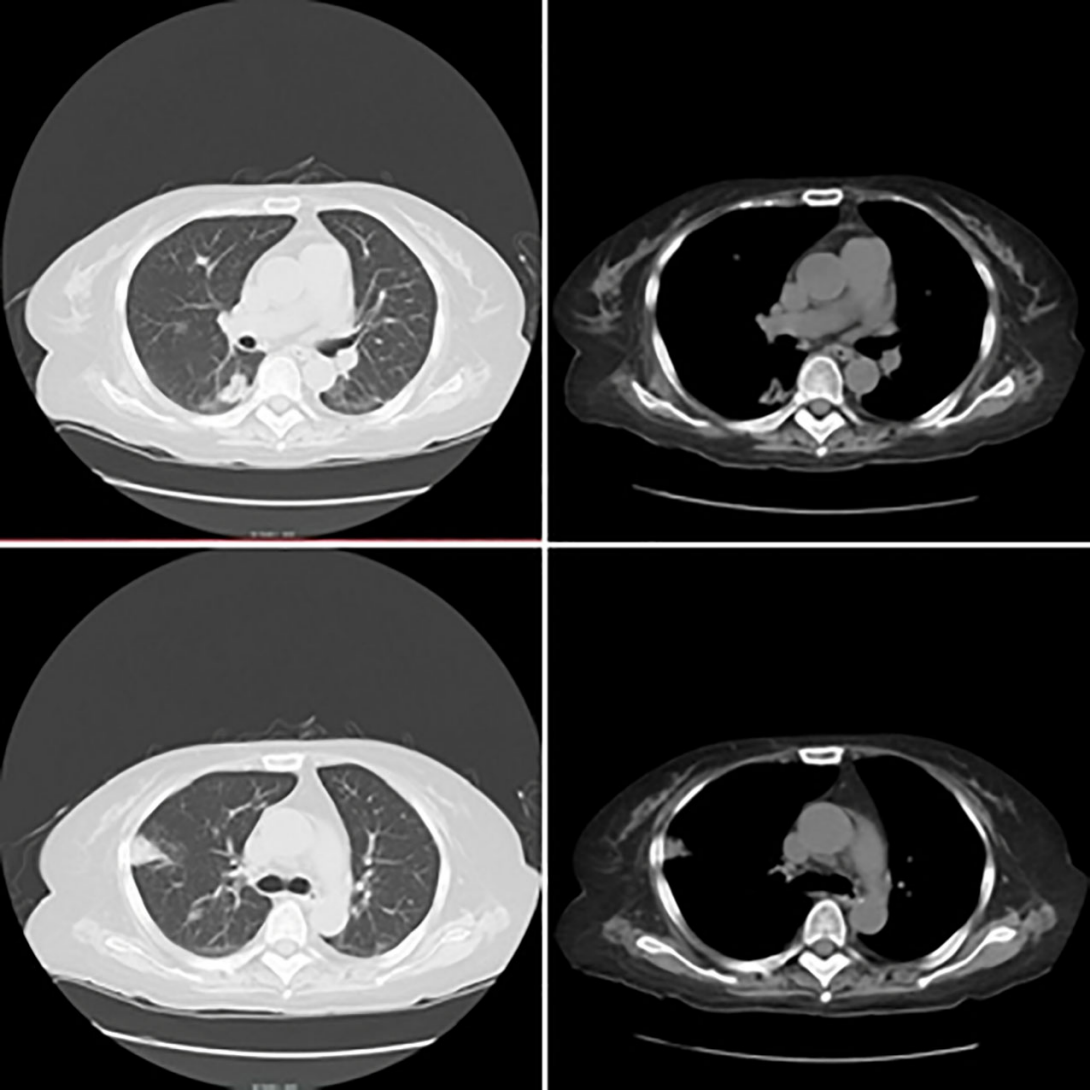
Chest CT scan before admission. The scan demonstrates bilateral interstitial pneumonia along with multiple pulmonary nodules and mass-like opacities. Comparison with the prior examination shows interval improvement and partial resolution of these opacities.

A subsequent chest CT after one month of treatment revealed interstitial pneumonia with some radiographic improvement compared to previous imaging (**Figure 1**). However, laboratory studies revealed pancytopenia: white blood cell count 2.4×10^9^/L, hemoglobin 84 g/L, platelet count 39×10^9^/L, along with a procalcitonin level of 0.288 ng/mL (normal range <0.05 ng/mL) and a significantly elevated NT-proBNP of 10,549 pg/mL. Due to the worsening pancytopenia and left orbital pain, she was transferred to our department for further evaluation and management.

Our physical examination of the patient showed a chronically ill appearance, decreased skin turgor, and left-sided proptosis with limited adduction and mild discharge. An old hyperpigmented rash was observed on the neck, along with hyperkeratotic rough skin on the deep aspects of both elbows and subcutaneous petechiae and ecchymoses over both forearms. Muscle strength and tone were normal.

**Figure 2:**
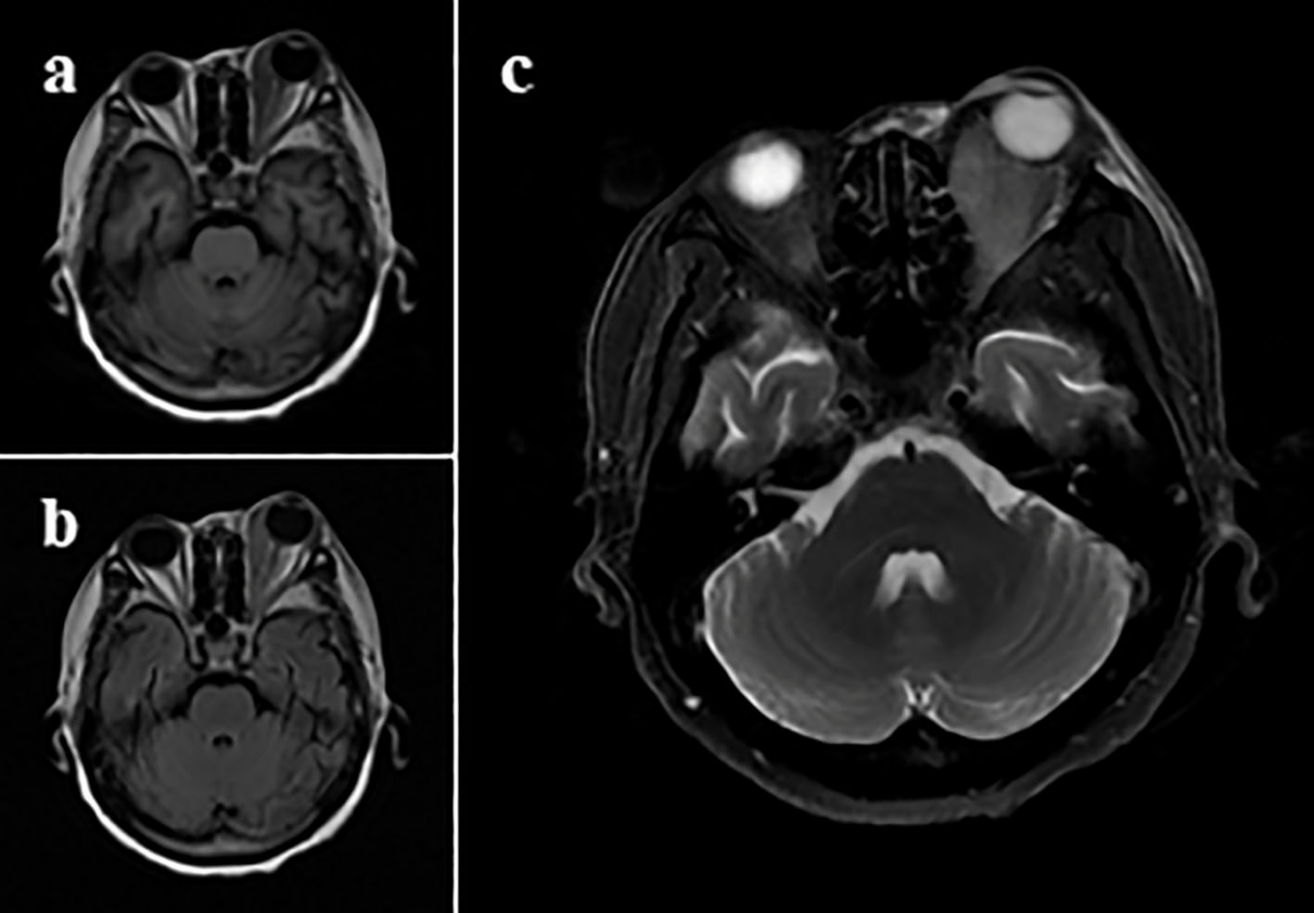
MRI of the head. T1 FLAIR **(a)**, T2 FLAIR **(b)**, and T2 FS FRFSE **(c)**. Images showed abnormal hyperintensity and enlargement of the left extraocular muscles (medial, superior, and inferior rectus), with associated mild proptosis, suggestive of an inflammatory pseudotumor.

Admission laboratory tests revealed a white blood cell count of 2.80×10^9^/L(normal 3.5-9.5×10^9^/L; neutrophils, 2.69×10^9^/L; lymphocytes, 0.04×10^9^/L; monocytes, 0.06×10^9^/L), hemoglobin 90 g/L (normal 130–175 g/L), platelet count 34×10^9^/L(normal 125-350×10^9^/L). Lymphocyte subset analysis showed a marked reduction in total T cells (CD3 + 67 cells/μL; normal 650–1880 cells/μL), including both helper T cells (CD3+CD4 + 53 cells/μL; normal 340–1072 cells/μL) and suppressor T cells (CD3+CD8 + 20 cells/μL; normal 210–742 cells/μL). B cells (CD3-CD19 + 6 cells/μL; normal 90–660 cells/μL) and NK cells (CD3-CD56 + 16 cells/μL; normal 46-590) were also severely decreased. Cytokine analysis demonstrated elevated levels of IL-6 (9.17 pg/mL; normal 0-5.3 pg/mL), IL-8 (35.49 pg/mL; normal 0-20.6 pg/mL), IL-10 (37.68 pg/mL; normal 0-4.91 pg/mL), and IFN-γ (13.73 pg/mL; normal 0-7.42 pg/mL). Autoimmune serology was positive for antinuclear antibody(1:320), anti-PL-12 antibody, and anti-SSB/Ro52 antibody. (**Figure 2**).

**Figure 3:**
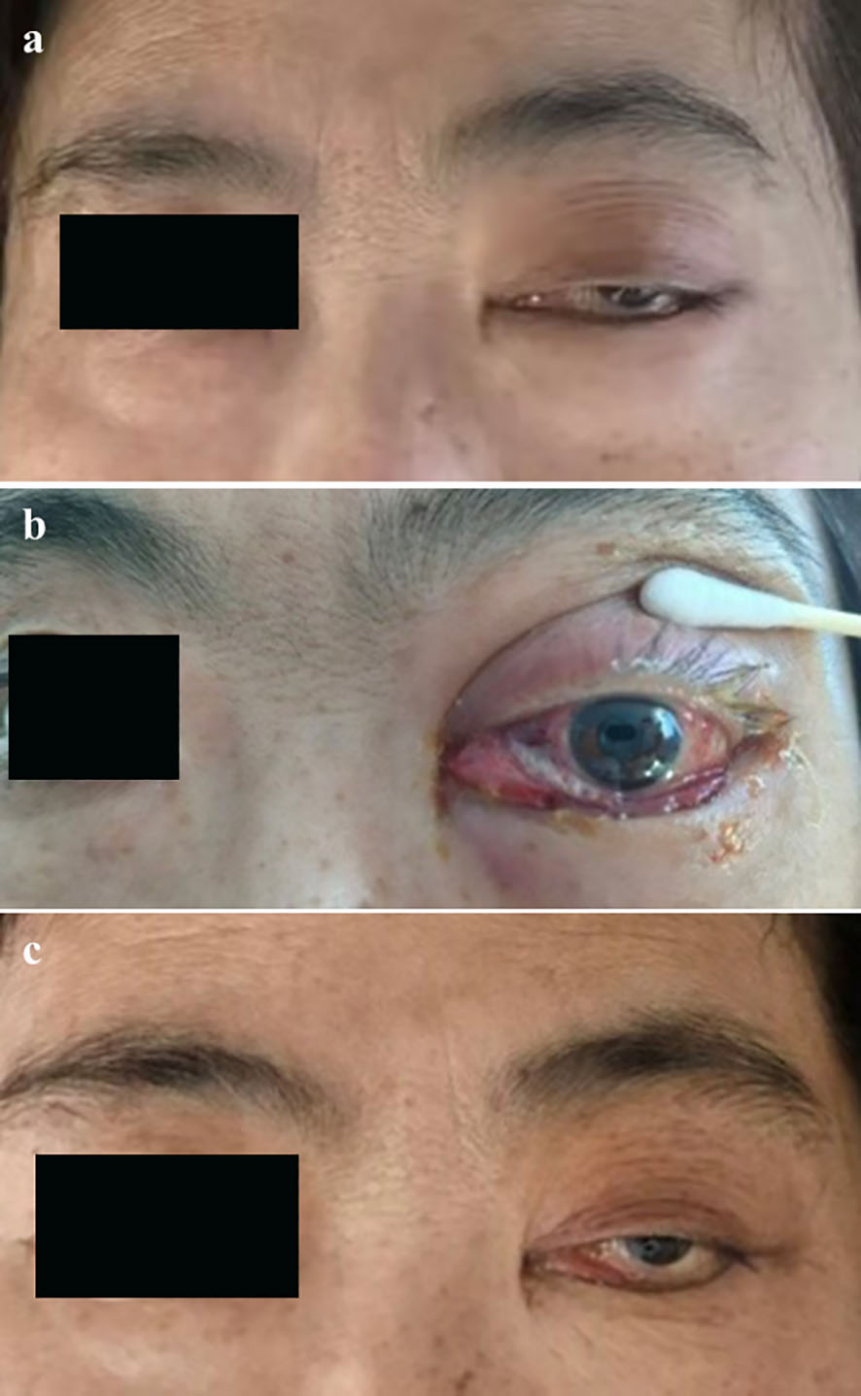
Serial orbital changes observed during the hospital course. Images were obtained at the following time points: **(a)** on admission, **(b)** 1 week after admission, and **(c)** 1 month after admission.

Based on clinical signs and laboratory findings, she was initially diagnosed with periorbital infection secondary to immunosuppression. Treatment included oral ganciclovir(0.5 g twice daily), oral TMP-SMZ(one tablet daily), intravenous piperacillin-sulbactam(4.5 g/8 h), intravenous voriconazole(0.2 g/12 h), intravenous methylprednisolone(40 mg twice daily), one session of peribulbar triamcinolone acetonide injection, and intravenous human immunoglobulin(10 g daily, for 5 days), combined with topical eye drops for symptomatic management. However, no improvement in pancytopenia was observed, and orbital swelling worsened. (**Figure 3**).

**Figure 4:**
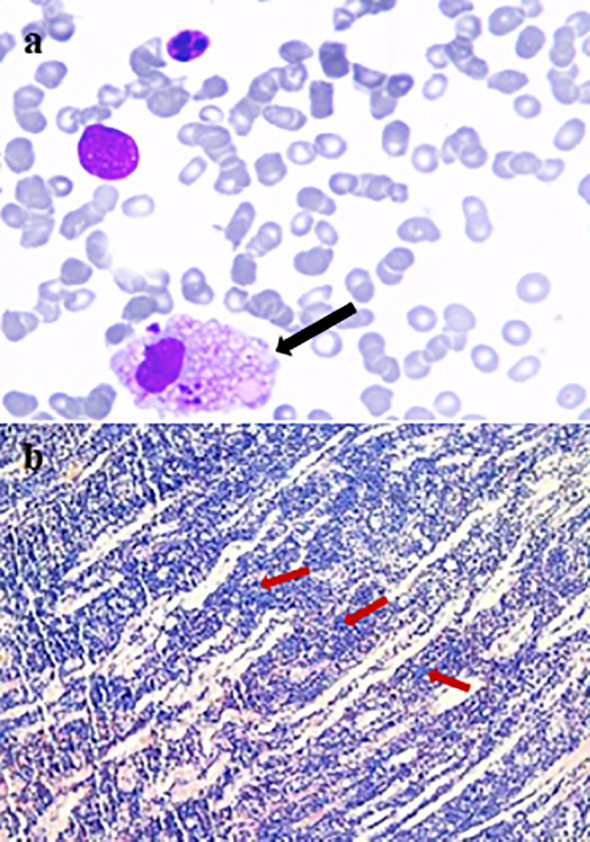
Pathological analyses. **(a)** Bone marrow aspirate. The smear shows hypoplasia and a platelet-producing megakaryocyte (black arrow). Myeloid-to-erythroid ratio is 3.39:1. Granulocytic series (69.5%) is normocellular and primarily consists of cells at or beyond the myelocyte stage. Erythroid series (20.5%) is normocellular with predominantly intermediate and late erythroblasts, without notable morphologic atypia. Lymphocytes constitute 9.5% of nucleated cells. Only three megakaryocytes are identified in the entire smear; platelets are rarely observed. **(b)** Extraocular muscle biopsy. (H&E, 100×) shows extensive interstitial infiltration by lymphoid cells (red arrow). Immunohistochemistry profile: CMV(–), CD3(+), CD20(–), CD68(–), CD5 (focal+), CD79a(–), CD21(–), Cyclin D1(focal+), SOX11(–), Bcl-2(focal weak+), Bcl-6(–), MUM1 (~70%+), CD23(–), CD10(–), Ki-67 (~90%+), CD2(+), CD7(+), CD4(focal +), CD8(focal +), CD56(+), Perforin(+), Granzyme B(+), TIA-1(+), CD30(+), ALK-1(–), CD38(focal +), P53(~60%+), C-myc(~40%+).

**Figure 5:**
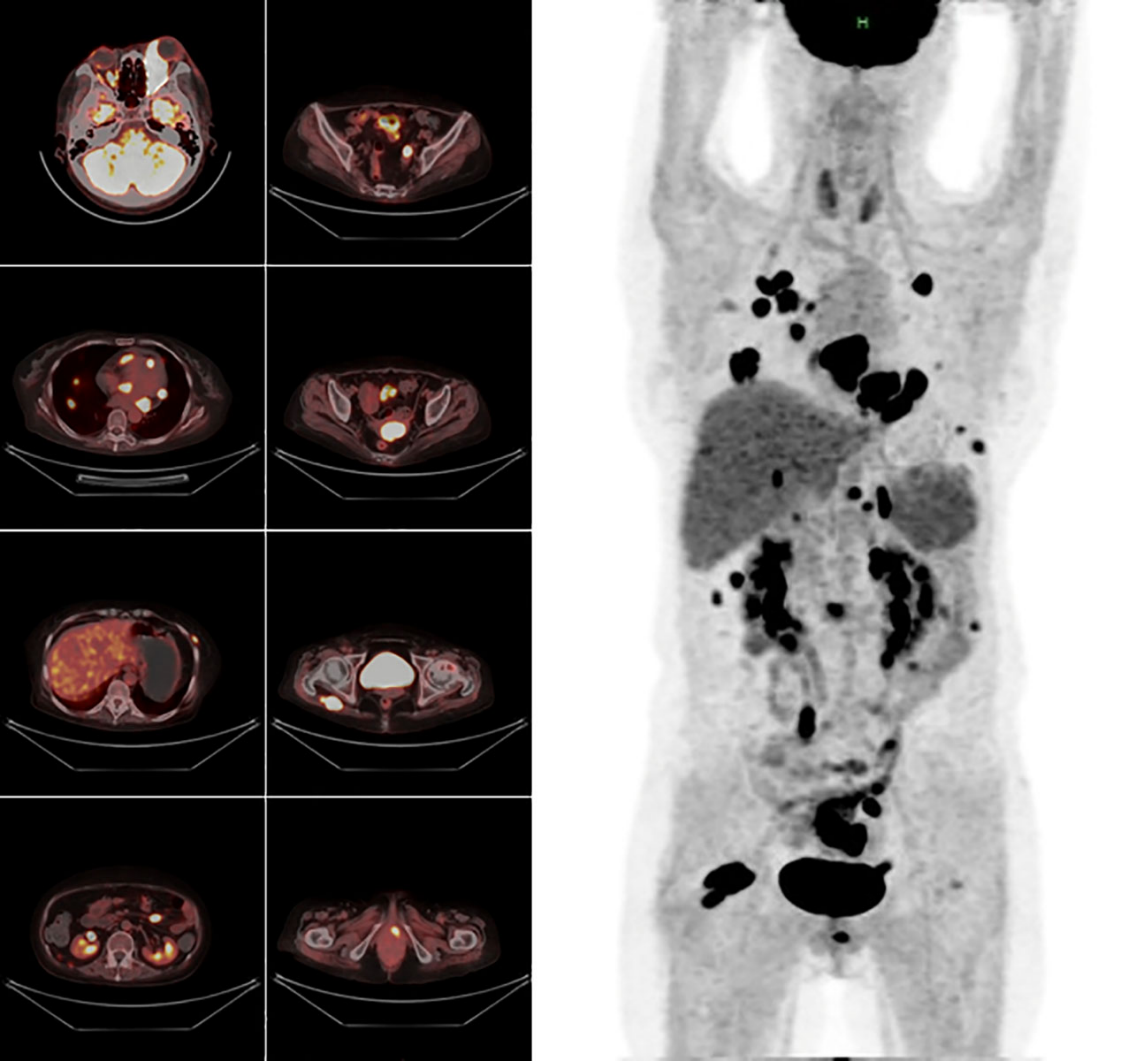
Whole-body PET/CT Scan. Hypermetabolism was noted in numerous muscles, lymph nodes, nodules, and visceral organs, indicating lymphoma with multisystem infiltration.

A multidisciplinary consultation was therefore conducted. Following recommendations, bone marrow aspiration and a biopsy of the orbital swelling were performed. The bone marrow smear showed hypoplasia, and the biopsy from the left extraocular muscle revealed interstitial infiltration by numerous atypical lymphoid cells with mitotic figures. EBER *in situ* hybridization was positive in most tumor cells, supporting a diagnosis of NK/T-cell lymphoma. Further evaluation with head and torso PET/CT imaging demonstrated widespread systemic lymphomatous involvement in multiple organs. (**Figures 4**, **5**).

## 3 Prognosis and follow-up

**Figure 6:**
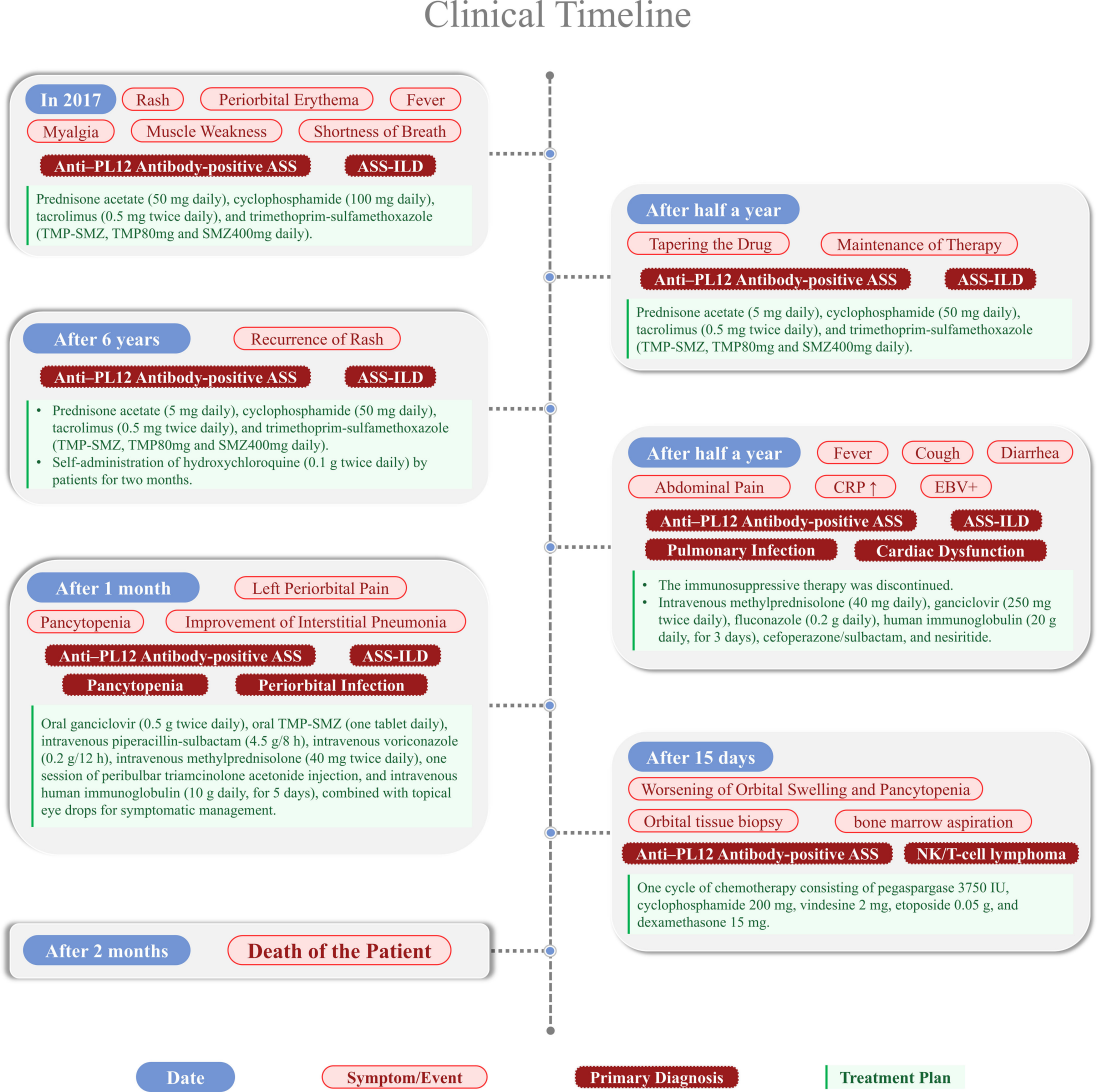
Patient case time.

Following a confirmed diagnosis of NK/T-cell non-Hodgkin lymphoma, she was transferred to the lymphoma oncology service. She received one cycle of chemotherapy consisting of pegaspargase 3750 IU, cyclophosphamide 200 mg, vindesine 2 mg, etoposide 0.05 g, and dexamethasone 15 mg. A second cycle was not administered. The patient passed away during a follow-up evaluation three months after discharge (**Figure 6**).

## 4 Discussion

IIM represents a heterogeneous group of disorders characterized by muscle weakness and inflammation. In 2017, the EULAR/ACR classification criteria incorporated clinical features, laboratory results, pathological findings, and imaging characteristics that identified distinct subgroups, including dermatomyositis(DM), polymyositis(PM), immune-mediated necrotizing myopathy(IMNM), inclusion body myositis(IBM), antisynthetase syndrome (ASS), and overlap myositis (8). ASS is one recognized subgroup of IIM (9), defined by muscle weakness, inflammatory muscle involvement, and the presence of specific autoantibodies. Patients with ASS commonly present with multi-system involvement affecting muscles, joints, lungs, and skin. The underlying pathogenesis is closely linked to immune dysregulation, where antisynthetase antibodies are believed to directly or indirectly provoke immune responses, resulting in systemic manifestations (10, 11).

The lungs are the most frequently involved extra-muscular organ in ASS, typically presenting as ILD. The spectrum of ILD ranges from inflammation to progressive fibrosis and scarring, and is associated with considerable morbidity and mortality, necessitating early recognition and immunosuppressive treatment. Patients with anti-PL-7 or PL-12 antibodies are more likely to experience acute symptom onset or progressive lung impairment compared to those with anti-Jo-1 antibodies. Furthermore, anti-PL-12-positive patients show a higher incidence of pleural effusion (10). In rapidly progressive or severe ILD, faster-acting medications such as intravenous cyclophosphamide (CYC) and corticosteroids (CS) are often the first-line choice. For less severe or slowly progressive cases, more conservative regimens are effective: mycophenolate mofetil (MMF), azathioprine (AZA), methotrexate (MTX), tacrolimus, or cyclosporine, with or without oral corticosteroids (12). In patients with progressive fibrosis, the antifibrotic agent nintedanib can help improve lung function (13). The risk of chronic and opportunistic infections should be considered before initiating treatment with disease-modifying antirheumatic drugs (DMARDs), immunosuppressants, and/or corticosteroids. Therapy should be guided by individual risk factors, with regular re-evaluation, appropriate screening, and prophylaxis for infections during treatment (14).

The patient’s initial diagnosis was established in 2017 at a specialized academic medical center. Upon detailed review of the original records, the diagnoses of anti-PL-12 antibody-positive ASS and ASS-ILD were confirmed based on the fulfillment of both the 2010 Connors and 2011 Solomon criteria. Critically, a comprehensive assessment at that time, including clinical presentation, serological tests, and laboratory data, revealed no evidence of an underlying malignancy. Treatment was initiated with full-dose glucocorticoids and maintained with immunosuppressants. Following this regimen, no pleural effusion, pulmonary hypertension, or progressive decline in pulmonary function was observed. During the current admission, imaging showed minimal progression of interstitial fibrosis compared to prior studies, and newly emerged pulmonary inflammation had resolved after treatment. This suggests that long-term combination therapy with glucocorticoids, cyclophosphamide, and tacrolimus effectively controlled the underlying ASS-ILD. During the current hospitalization, severe reductions in all lymphocyte subsets were observed, which may be attributed to the long-term combination therapy over the past 7 years. This regimen likely suppressed T-cell activation and induced apoptosis, leading to marked decreases in T-lymphocyte subsets. The persistent immunosuppression significantly compromised the patient’s immune surveillance against viruses. Blood tests obtained after admission revealed a notably elevated EBV load. She was eventually diagnosed with NK/T-cell non-Hodgkin lymphoma with distant metastasis. This case raises discussion regarding the relationship between viral infection and lymphomagenesis.

Current studies indicate that patients with IIM have a significantly increased risk of malignancy (15, 16). Among the disease subtypes, DM carries the highest risk of associated malignancy, while PM and IBM are associated with a comparatively lower risk (17, 18). Cases of hematologic malignancies occur more frequently in DM/PM patients and are diverse in type (19). However, these studies did not specifically mention the risk of malignancy in patients with ASS. DM shows stronger associations with ovarian, lung, pancreatic, gastric, colorectal cancers, and non-Hodgkin lymphoma, whereas PM is more frequently associated with lung, bladder cancer, and non-Hodgkin lymphoma (20). A retrospective analysis of 32 PM/DM patients with hematologic malignancies revealed that lymphomas (81.2%) were predominantly B-cell lymphomas (62.5%), followed by T-cell lymphomas (12.5%) and Hodgkin lymphoma (6.3%) (19). All these patients tested negative for anti-PL-12 antibodies. The perceived low cancer risk associated with ASS has been systematically reassessed by recent epidemiological studies. Multiple cohort studies have demonstrated a significantly increased overall cancer risk in patients with ASS, with a standardized incidence ratio (SIR) of 5.4. Furthermore, 11.4% to 17.0% of patients developed malignancies within 5 years before or after diagnosis. The slightly higher incidence observed in the postdiagnosis period suggests that both the underlying disease process and its subsequent treatment may contribute to tumorigenesis (21). Regarding the spectrum of cancer types, hematologic malignancies appear to be relatively uncommon in patients with ASS. Existing literature is largely confined to case reports, which have documented instances of B-cell lymphoma (22) and T-cell lymphoma (23) in patients positive for anti-PL-7 antibodies. A particularly instructive case involved the development of EBV-associated lymphomatoid granulomatosis in the context of azathioprine therapy, which subsequently progressed to diffuse large B-cell lymphoma (24). This progression suggests a synergistic oncogenic effect between an immunosuppressed state and EBV reactivation. These findings further suggest that the pathogenesis of ASS-associated malignancies involves a complex interplay of multifactorial mechanisms. Specifically, prolonged immunosuppressive therapy may lead to an imbalance in immune cell subsets, while patients inherently exhibit defects in immune surveillance. Concurrently, the persistent activation of the TNF pathway due to chronic inflammation likely contributes to a pro-cancer microenvironment. Together, these factors may synergistically promote tumorigenesis (21).

The pathogenesis of non-Hodgkin lymphoma (NHL) remains incompletely understood. However, it is established that disordered immune responses under conditions of immunosuppression or autoimmune disease may contribute to NHL development. Previous studies have reported an elevated risk of NHL in patients with systemic lupus erythematosus, rheumatoid arthritis, and primary Sjögren’s syndrome. Analysis of medication use in these patients suggests that methotrexate may be associated with the occurrence of NHL (25). Currently, immunodeficiency is considered one of the strongest known risk factors for NHL (26).

Natural killer/T-cell lymphoma (NKTCL) is a rare subtype of non-Hodgkin lymphoma that shows geographic variation, with higher prevalence in Asia and Latin America. It is classified as an EBV-associated lymphoproliferative disorder (27). Epstein-Barr virus, also referred to as human herpesvirus 4 (HHV-4), contains a double-stranded DNA genome of approximately 172 kbp and belongs to the gammaherpesvirus subfamily. This widespread virus infects over 95% of the global population, and the primary infection is typically asymptomatic (28). EBV establishes lifelong latency by infecting and persisting in memory B cells. In immunocompromised individuals, impaired immune surveillance can lead to viral reactivation and the development of life-threatening conditions (29). EBV is involved in the pathogenesis of several lymphomas, including extranodal natural killer/T-cell lymphoma (NKTCL), Burkitt lymphoma (BL), and Hodgkin lymphoma (HL) (30). The virus exhibits three distinct latency patterns based on its gene expression profile. In nearly all NKTCL cases, EBV infection is detectable, and most patients demonstrate latency type II, which is characterized by expression of EBNA1, BART, LMP1, LMP2A, and LMP2B (31). EBV promotes tumor initiation and progression through direct pathways, such as inducing genomic instability, regulating oncogene and tumor suppressor gene expression, and interfering with cell proliferation, cell cycle, and apoptosis, as well as indirect mechanisms, including immunosuppression, metabolic reprogramming, and chronic inflammation (32).

Several observations from this case provide insight into the pathogenesis of lymphoma in the setting of ASS. The patient’s history of exposure to a long-term, high-intensity multidrug regimen may have contributed to lymphomagenesis. At the time of current admission, immunosuppressive agents had been discontinued, and the patient presented with a high EBV DNA load. Following initiation of a regimen comprising corticosteroids, antiviral therapy, and cytotoxic agents, the EBV DNA load decreased to 8.74×10^3^ copies/mL, demonstrating a downward trend. In this patient, prolonged immunosuppression was likely a critical factor in EBV reactivation, and this chronic EBV infection may, in turn, have contributed to the subsequent development of NKTCL.

This case offers several insights into the pathogenesis of lymphoma in patients with ASS receiving long-term immunosuppressive therapy. The patient’s exposure to a prolonged, high-intensity multidrug regimen likely induced an immunocompromised state that facilitated EBV reactivation, as evidenced by a high EBV DNA load at presentation. The subsequent decline in viral load (from 2.25×10^4^ copies/mL to 8.74×10^3^ copies/mL) after initiation of withdrawal of immunosuppressive agents and antiviral and cytotoxic therapy further supports the role of immunosuppression in facilitating viral replication. Collectively, these observations suggest a pathogenic sequence in which prolonged immunosuppression promotes EBV reactivation, and the resulting chronic EBV infection may contribute to the development of NKTCL. This case highlights the importance of EBV surveillance in ASS patients undergoing long-term intensive immunosuppression.

## 5 Conclusion

At present, research on ASS complicated by malignancy remains relatively limited. Most studies focus on anti-Jo-1 antibody-positive populations, while the literature regarding anti-PL-12 antibody-positive patients is sparse. Long-term maintenance therapy exerts a persistent influence on the immune system. According to the 2022 EULAR recommendations, patients with autoimmune inflammatory rheumatic diseases (AIIRD) receiving antirheumatic therapy should undergo regular screening for tuberculosis, hepatitis B, hepatitis C, and HIV. However, specific screening intervals and recommendations for other pathogens have not been clearly established (14). This case highlights the importance of maintaining vigilance during long-term antirheumatic treatment. It is essential to enhance follow-up and immune function monitoring, conduct regular screening for infections—including less common viruses such as EBV—and promptly initiate malignancy screening when disease progression occurs to ensure early diagnosis and intervention.

## Data Availability

The original contributions presented in the study are included in the article/supplementary material. Further inquiries can be directed to the corresponding author.
